# The pediatric oncology exercise field speeds up to address important issues regarding chemotherapy-related cardiotoxicity

**DOI:** 10.3389/fped.2022.998337

**Published:** 2022-10-14

**Authors:** Maxime Caru, Daniel Curnier

**Affiliations:** ^1^ Department of Pediatric, Division of Hematology and Oncology, Penn State College of Medicine, Hershey, PA, United States; ^2^ Department of Public Health Sciences, Penn State College of Medicine, Hershey, PA, United States; ^3^ School of Kinesiology and Physical Activity Sciences, Faculty of Medicine, University of Montreal, Montreal, QC, Canada; ^4^ Division of Pediatric Hematology and Oncology, Sainte-Justine University Health Center, Research Center, Montreal, QC, Canada

**Keywords:** physical activity, prevention, cardio-oncology, Childhood Cancer Survivors, survivorship, doxorubicin-related cardiotoxicity, healthy lifestyle, cardiovascular risk factors

## Introduction

Chemotherapy-related cardiotoxicity is well understood and recognized in oncology (1, 2). Randomized controlled trials have shown that exercise has the potential to prevent chemotherapy-induced cardiotoxicity in adults diagnosed with cancer (3–6). Nevertheless, the pediatric oncology exercise field is in emergence comparatively to the adult oncology exercise field and has also the potential to adequately respond to the chemotherapy-related cardiotoxicity burden that several childhood cancer survivors face. Chemotherapy-related cardiotoxicity causes multiple late comorbidities and is the most common cause of late-onset post-treatment morbidity and mortality in childhood cancer survivors (7). In fact, childhood cancer survivors experience a 2- to 10-fold increased risk of cardiovascular diseases relative to the general population (8) and are seven times more likely to die from cardiovascular complications (e.g., high-grade ectopy, impaired left ventricular contractility, late congestive heart failure, and sudden death) (9, 10). Through the recently published international pediatric oncology exercise guidelines, clinical and research experts in pediatric oncology have highlighted that “cardiotoxicity” is a specific condition that needs to be considered in the field of pediatric oncology exercise (11). Hence, in a recent scoping review discussing the benefits of exercise at different intensities to prevent and manage cancer treatment-related cardiac dysfunction in childhood and adolescent cancer survivors, authors pointed out the necessity to do clinical research to consolidate current findings (12). This opinion paper discusses the potential of exercise for childhood cancer survivors to prevent chemotherapy-related cardiotoxicity and associated cardiovascular risk factors. It also presents the primary key challenges encountered in cardio-oncology and exercise settings.

## Cardiac health and exercise capacity in childhood cancer survivors

Every year, almost 10,500 children receive a diagnosis of cancer in the United-States. Among them, approximately 85% live at least 5 years post-diagnosis. As of 2019, the National Cancer Institute has reported that there are 483,000 childhood cancer survivors in the United-States (13). Several childhood cancer survivors are facing chronic health problem related to their cancer treatments. A study combining the Childhood Cancer Survivor Study (CCSS) and the Surveillance Epidemiology and End Results (SEER) data reported that ∼70% of childhood cancer survivors face mild to moderate chronic condition, and 32% of them face a severe, disabling or life-threatening chronic condition (14). The authors also showed that chronic health problems increase with age no later than 5 years post-diagnosis (14).

Although medical research and progress have allowed to reach better survival rates, children’s exposure to chemotherapeutic agents causes several long-term adverse effects, such as cardiovascular diseases (15). Studies have observed that survivors exposed to anthracycline drugs may face subclinical dysfunctions and cardiac abnormalities (16–21). Indeed, some childhood cancer survivors have a reduced ejection fraction and impaired left ventricular contractility. Moreover, they are at high risk of developing cardiomyopathy and researchers have shown that some childhood cancer survivors are at an early stage of heart failure a few years after the end of their treatment (20, 22). Chemotherapy-related cardiotoxicity is a leading cause of morbidity and mortality by late congestive heart failure and sudden death (9, 10). Findings from the CCSS have shown that they are seven times more likely to die from late cardiovascular complications than their healthy peers (23).

As a result of the treatment, they received during their cancer, childhood cancer survivors have a lower exercise capacity and cardiorespiratory fitness than their healthy peers (24–26). A study performed as part of the St Jude Lifetime Cohort Study (1,041 people who had survived cancer ≥10 years) showed that exercise intolerance [defined by the authors as the inability to perform as expected on a measure of exercise capacity (<85% of predicted VO2 peak and maximal aerobic capacity <7.9 metabolic equivalents)] was associated with mortality (HR = 3.9; 95% CI = 1.09–14.14), global longitudinal strain (OR = 1.71; 95% CI = 1.11–2.63) and chronotropic incompetence (OR = 3.58; 95% CI = 1.75–7.31) (25). Global longitudinal strain is a sensitive measure to detect subclinical left ventricular dysfunction in patients at high risk for heart failure (25, 27). Chronotropic incompetence, however, is a predictor of mortality in cardiac patients and has been associated with cardiac autonomic nervous system dysfunction (28–31). Evidence from observational studies shows that these parameters can be improved by exercise in those who are physically active compared to those who are inactive (32, 33). Indeed, in 15,450 adult survivors of childhood cancer, a multicenter cohort analysis observed that exercise exposure was significantly associated with a reduction in the cumulative incidence of all-cause mortality, relapse and health-related mortality (34). A large case-control study showed that exercising less than 3 days per week was associated with an increased risk for all-cause mortality, compared to childhood cancer survivors who exercised more than 3 days per week (35).

## Healthy lifestyle in childhood cancer survivors

Over the past decade, clinical and research experts in pediatric oncology have demonstrated that exercise is beneficial and safe for childhood cancer survivors. A state-of-the-art review recently highlighted the importance of exercise in childhood cancer survivors and its key role in preventing chemotherapy-related cardiotoxicity (15). Indeed, chemotherapy-related cardiotoxicity leads to cardiac dysfunctions (e.g., decline in left ventricular ejection fraction), because of mitochondrial biogenesis impairment (36), whereas exercise stimulates mitochondrial biogenesis (37). Moreover, observational studies have reported that exercise in childhood cancer survivors improves left ventricular ejection fraction (38, 39) and markers of cardiovascular health (33, 40). Several other pathways also need to be taken into consideration as potential mechanisms of chemotherapy-induced cardiotoxicity, such as inhibition of topoisomerase 2B, oxidative stress, DNA damage and Ca^2+^ release and myocardial fibrosis (41–44). Nevertheless, research on the impact of exercise on theses parameters needs to be pursued in human and animal models to strengthen the evidence (45, 46). Studies have shown, however, that chemotherapy-induced cardiotoxicity may not be reversible in animal models and observational human studies biopsies (47–49) which reinforces the importance of exercising during cancer treatments.

Nevertheless, few childhood cancer survivors are physically active. The American Association for Cancer Research (AACR) has reported that almost one in two survivors are not following the physical activity guidelines and that >70% are less likely to be physically active than their healthy peers (50). In an observational cohort study of childhood acute lymphoblastic leukemia survivors, it has been reported that survivors and healthy people had a clinically equivalent level of moderate to vigorous physical activity per week, despite a substantially lower cardiorespiratory fitness level in survivors (24). Long-term follow-up of childhood cancer survivors has shown that their physical activity level worsens significantly until they ultimately reach a sedentary behavior state (51). These findings are worrying because a low physical activity level is associated with lower cardiac health (33, 35) and an inactive lifestyle has been associated with cardiovascular disease in childhood cancer survivors (52). To enhance cardiac health prognoses and favor health-related benefits in cancer survivors, the American College of Sports Medicine (ACSM) guidelines recommend achieving at least 30 min per day of aerobic exercise training, in addition to doing resistance training (53). Evidence has shown promising results on cardiac function, while requiring further studies to explore the preventative effects of exercise on cardiotoxicity (41, 53, 54).

Considering the current literature, it is important to note that in several childhood cancer survivors, chemotherapy-related cardiotoxicity is reinforced by their lifestyle and behavioral risk factors adopted during and after their treatment (55, 56). It has been well demonstrated that physical inactivity and sedentary behavior favor cardiovascular diseases (57). As an example, physical inactivity has been described as the fourth cardiovascular risk factor and was pointed out to be responsible for over 5 million deaths (58, 59). Cardiovascular diseases are also associated with other risk factors, such as depressive symptoms, metabolic syndrome, weight status (abdominal obesity, overweight, obesity), dyslipidemia, diabetes, hypertension and smoking (60). These cardiovascular risk factors are commonly reported in childhood cancer survivors (61, 62). Thus, addressing childhood cancer survivors' cardiovascular risk factors could be a powerful way to prevent chemotherapy-related cardiotoxicity, in addition to adopting a preventative approach initiated at cancer diagnosis (63, 64).

## Primary key challenges

In a recent scoping review, Wogksch et al. described the associations between physical activity and chronic diseases and reported that childhood cancer survivors who engage in physical activity decrease their risk of cardiovascular diseases, in addition to improving markers of cardiovascular diseases and their risk of mortality, compared to childhood cancer survivors who do not engage in physical activity (33). These findings confirm those of Slater et al., who reported in a cross-sectional study that childhood cancer survivors (*n* = 319) with a high physical activity level had lower percent fat mass, abdominal subcutaneous and visceral fat, and greater lean body mass and insulin sensitivity than survivors who reported having a low physical activity level (65). Considering the current literature in cardiology, physical activity has a prophylactic effect on cardiovascular risk factors and does not require for patients to engage in several hours per day of exercise (66). Several studies share the important message that physical inactivity always needs to be avoided in childhood cancer survivors, which can be achieved by engaging in regular physical activity. This also joins the international pediatric oncology exercise guidelines global message that moving is important for all pediatric patients with cancer and survivors (11).

Exercise can be a useful approach to address chemotherapy-related cardiotoxicity in pediatric patients considering that the probability to develop a cardiovascular disease is approximately 3% in childhood cancer survivors who are 30 years old, a number that increases to about 10% at 45 years old (67). The literature highlights that exercise can be an even more powerful approach when addressing cardiovascular risk factors that may have an impact on chemotherapy-related cardiotoxicity observed in childhood cancer survivors. Managing risk factors in cardio-oncology can be challenging considering that most of childhood cancer survivors are under several cardiac parameter thresholds. It is also important to remind that the cardiac challenges they face are different than those in adult patients with cancer or cardiac patients. During the first year of their follow-up, childhood cancer survivors do not have more cardiovascular risk factors than their healthy counterparts (68). While the clinical observations could be encouraging at first glance, long-term follow-ups have shown that the prevalence of cardiovascular risk factors increases with time, as well as the risk of cardiovascular diseases (67, 69). For example, an observational study showed that the impact of chemotherapy treatment was subclinical and that childhood acute lymphoblastic leukemia survivors were in the first stage of heart failure (20). The authors hypothesized that these survivors were compensating for subclinical cardiac remodeling. These findings emphasize the importance of providing cardio-oncology follow-ups to survivors exposed to chemotherapy treatments even if they are not at risk of cardiac diseases at the time of their follow-up appointment. Therefore, ongoing studies are being conducted to enhance our understanding of the effect of exercise on cardiotoxicity in childhood cancer survivors, such as the HIMALAYAS Trial (NCT05023785). The HIMALAYAS trial aims to evaluate the impact of exercise on cardiovascular health in childhood, adolescent and young adult cancer survivors, and is a good example to understand how the research field of pediatric oncology exercise is evolving.

Another challenge is the diagnosis of cardiac dysfunction in childhood cancer survivors. There is no broad consensus on how cardiotoxicity should be measured in childhood cancer survivors, leading to heterogeneity between studies (70–74). Indeed, cardiotoxicity can be measured by a combination of different parameters, such as reduced resting systolic function, reduced resting diastolic dysfunction, impaired hemodynamics and systolic functional reserve measured during exercise or reduced exercise capacity or cardiopulmonary fitness (VO_2_ peak). In this sense, clinicians and researchers need to be cautious when interpreting the reported data and findings on the associations between exercise and cardiotoxicity.

While cardiac dysfunctions can be observed at rest, research experts and clinicians have started recommending an exercise stress test coupled with cardiac imaging techniques to unmask cardiac impairments that are not observable at rest in cancer survivors (75–77). It has been demonstrated that survivors can achieve a safe maximal cardiopulmonary exercise test without being limited by symptoms, potential overprotection, or musculoskeletal issues (78). Nevertheless, in clinical settings, performing a maximal cardiopulmonary exercise test can be a challenge since it requires human and financial resources. And although there are other exercise testing procedures that can be used (79), a maximal cardiopulmonary exercise test remains the gold standard in exercise physiology to measure the maximal oxygen consumption of childhood cancer survivors and their cardiorespiratory fitness, which is associated to their cardiac health. It is also important to note that not all exercise physiologists or healthcare providers are trained to perform a maximal cardiopulmonary exercise test and that not all countries authorize an exercise physiologist to perform this type of maximal exercise test, even under the supervision of a cardiologist.

Finally, to strengthen the pediatric oncology exercise field to address the cardiac health of children with cancer and childhood cancer survivors, exercise professionals should be trained to be experts in oncology and more specifically in cardio-oncology, as recommended (80–82). Having expertise in these two major fields can be a real challenge since a number of factors have to be taken into consideration. For example, evidence-based medicine in pediatric cardio-oncology is different than in adult cardio-oncology. In both cases, it is important to consider patients' health status, treatment trajectory and adopt a multidisciplinary approach.

## Conclusion

The pediatric oncology exercise field is in its early stages and future studies are required to explore the benefits of exercise on the cardiovascular health of childhood cancer survivors. As discussed, the evidence regarding chemotherapy-related cardiotoxicity is limited and looking at the big picture by addressing cardiovascular risk factors would be a research path to follow in order to improve our knowledge. It is also important to take into consideration that childhood cancer survivors are children, adolescents and adult patients with different needs and who are at different stages of their physiological and cardiac development. Addressing chemotherapy-related cardiotoxicity is a great challenge and we hope that this paper will generate interest and ideas.

## Author contributions

MC designed and wrote the manuscript, and DC revised and contributed to the writing of the manuscript. All authors contributed to the article and approved the submitted version.
